# Construction of ThermoMaze

**DOI:** 10.21769/BioProtoc.5044

**Published:** 2024-08-05

**Authors:** Aryeh Rothstein, Mihály Vöröslakos, Yunchang Zhang, Kathryn McClain, Roman Huszár, György Buzsáki

**Affiliations:** 1 Neuroscience Institute, School of Medicine, New York University, New York, NY, USA; 2 Department of Neurology, School of Medicine, New York University, New York, NY, USA

**Keywords:** Rodent behavior, Electrophysiology, Thermoregulation, Sharp-wave ripples

## Abstract

Physiological changes during awake immobility–related brain states remain one of the great unexplored behavioral states. Controlling periods of awake immobility is challenging because restraining the animal is stressful and is accompanied by altered physiological states. Here, we describe the ThermoMaze, a behavioral paradigm that allows for the collection of large amounts of physiological data while the animal rests at distinct experimenter-determined locations. We found that the paradigm generated long periods of immobility and did not alter the brain temperature. We combined the ThermoMaze with electrophysiology recordings in the CA1 region of the hippocampus and found a location-specific distribution of sharp-wave ripple events. We describe the construction of the ThermoMaze with the intention that it helps enable large-scale data recordings on immobility-related brain states.

Key features

• Controlling periods of awake immobility in rodents.

• Electronic-friendly analog of the Morris water maze.

## Background

Neural oscillations operate as the language of the brain, underlying neuronal processes such as cognitive capabilities [1]. Recent advances (e.g., optogenetics) have enabled researchers to explore in detail the anatomy and functioning of the brain irrespective of behavior [2]. However, utilization of behavioral paradigms is critical for understanding the relationship between behavioral states and ongoing brain activity [3]. In basic neuroscience studies, behavioral paradigms should utilize natural stimuli and investigate behaviors that are part of the animal’s ethogram [4].

One ubiquitous rodent behavioral task is the Morris water maze (MWM), in which a rodent (generally a rat or mouse) needs to swim through a pool to find a platform to stand on [5]. The periods of immobility on the platform provide important neural information, as periods of wakeful rest (similar to periods of immobility) have been linked to initial consolidation processes with increased replay in humans [6]. Despite this and its abundant use, the MWM is a stressful environment for both mice [7] and rats [8] and, as such, does not serve as a natural environment for these rodents. Furthermore, the aqueous environment of the maze makes electrical recordings difficult, if not impossible, hindering the ability of scientists to dissect occurring physiological changes.

Here, we describe the construction of an electronic-friendly analog of the MWM, the ThermoMaze. The paradigm was validated via electrophysiological measures, but its use extends any electronics-based data collection modality, including but not limited to freely moving 2-photon imaging, wide-field calcium imaging, and fiber photometry. Utilizing an abrasively cold environment with specific hotspots, the paradigm capitalizes on thermotaxis and thermoregulatory behaviors of mice to generate long periods of immobility during the behavioral task, enabling a deeper and richer understanding of the immediate oscillatory changes underlying memory.

## Materials and reagents

*Note: Information on vendors, model numbers, and links to purchase equipment is provided when possible. The list of materials used here can also be accessed at* *https://github.com/misiVoroslakos/3D_printed_designs/blob/main/ThermoMaze/BOM_ThermoMaze_v03.txt*

1. Peltier elements (Amazon, catalog number: TEC1-12706, https://www.amazon.com/GeeBat-TEC1-12706-Thermoelectric-Heatsink-Cooling/dp/B01IT8SAZG/)
2. Aluminum water cooling block (Amazon, catalog number: B08JKP6HYC, https://www.amazon.com/uxcell-Aluminum-Heatsink-Computer-Graphics/dp/B08JKP6HYC/)
3. Submersible water pump (Amazon, catalog number: B085NQ5VVJ, https://www.amazon.com/LEDGLE-Submersible-Ultra-Quiet-Dual-Purpose-Hydroponics/dp/B085NQ5VVJ/)
4. UV-resistant cast acrylic 1/8” thickness, white (McMaster, catalog number: 8505K743, https://www.mcmaster.com/8505K743)
5. Clear soft PVC plastic tubing for air and water, 5/16" ID, 7/16" OD (McMaster, catalog number: 5233K59, https://www.mcmaster.com/5233K59/)
6. Thermally conductive epoxy (MG Chemicals, catalog number: 8349TFM-25ML, https://www.amazon.com/MG-Chemicals-8349TFM-Thermally-Condcutive/dp/B08Z73HH23/)
7. Wood epoxy
8. 3D-printed frame (https://github.com/misiVoroslakos/3D_printed_designs/tree/main/ThermoMaze)
9. K-type thermocouple (OMEGA, catalog number: 5TC-TT-K-40-36, https://www.newark.com/omega/5tc-tt-k-40-36/thermocouple-wire-type-k-40awg/dp/30AC8682)
10. Thermistor (Mouser, catalog number: 954-223FU3122-07U015, https://www.mouser.com/ProductDetail/Semitec/223Fu3122-07U015/?qs=raqtESnDWsAF5g197HBRcQ%3D%3D)
11. Crushed ice
12. Refreezable ice packs
13. Bucket for water
14. FLIR C5 compact thermal imaging camera (Mouser, catalog number: 685-89401-0202, https://www.mouser.com/ProductDetail/Teledyne-FLIR/89401-0202?qs=vmHwEFxEFR8KCgYmUURHLQ%3D%3D)

## Equipment

1. Video camera (Basler, model: ace 2 a2A2590-60ucBAS)
2. Lens (Basler, 16 mm, model: C23-1616-2M)
3. GPIO cable (Basler, model: GP-I/O Cable 6p/open, 10 meters)
4. Tripod mount (Basler, catalog number: 2200000314)
5. E36102A benchtop current generator (Keysight Technologies, catalog number: E36102A/0E3/902, https://www.mouser.com/ProductDetail/Keysight/E36102A-0E3-902?qs=YCa%2FAAYMW03ip5pRT6eiig%3D%3D)
6. Microcontroller circuit board (Arduino, model: Mega 2560 Rev3, https://store-usa.arduino.cc/collections/boards-modules/products/arduino-mega-2560-rev3?_pos=2&_fid=04e83805a&_ss=c)
7. 8-Channel relay module (Amazon, catalog number: 101-70-102, https://www.amazon.com/SainSmart-101-70-102-8-Channel-Relay-Module/dp/B0057OC5WK/ref=asc_df_B0057OC5WK/?tag=hyprod-20&linkCode=df0&hvadid=312070784062&hvpos=&hvnetw=g&hvrand=14266163912783471503&hvpone=&hvptwo=&hvqmt=&hvdev=c&hvdvcmdl=&hvlocint=&hvlocphy=9067609&hvtargid=pla-348881239402&th=1)
8. Microcontroller circuit board (Arduino, model: Uno)
9. Red LED (Amazon, catalog number: ED_P05_R_100Pcs, https://www.amazon.com/EDGELEC-100pcs-Resistors-Included-Emitting/dp/B077XDYTTP)
10. Resistor (470 Ohm, catalog number: ED_P05_R_100Pcs, https://www.amazon.com/EDGELEC-100pcs-Resistors-Included-Emitting/dp/B077XDYTTP)
11. Cables for Arduino (ELEGOO, model: EL-CP-004, https://www.amazon.com/Elegoo-EL-CP-004-Multicolored-Breadboard-arduino/dp/B01EV70C78)
12. Banana plugs with micro grabbers (Pomona, catalog number: EM5053-12-0#)
13. Handheld thermometer (Omega, model: HH800, https://www.omega.com/en-us/test-inspection/handheld-meters/temperature-and-humidity-and-dew-point-meters/p/HH800)

## Software and datasets

1. Arduino IDE (version 1.8.19, open source). This was used to control the submerged DC pumps and to run the behavioral paradigm
2. Intan RHD2000 USB Interface Board software was used to test the electrical collections and collect data
3. Pylon Viewer (Basler AG, Ahrenberg, Germany; version 6.2.0, available at https://www2.baslerweb.com/en/downloads/software-downloads/#version=6.2.0) was used to control the video camera from the computer.
4. Windows built-in video recorder was used to record infrared (IR) video from the FLIR camera
5. MATLAB (version R2021A) was used to analyze data using custom scripts

**Key links:**

1. Important information on using 3D printing files can be found at: https://github.com/misiVoroslakos/3D_printed_designs/blob/main/ThermoMaze/README.md

2. The Arduino code for running the behavioral paradigm can be found here: https://github.com/misiVoroslakos/3D_printed_designs/blob/main/ThermoMaze/ThermoMaze_control/Cooling_box_v04_relays_20220410.ino

3. The file for 3D printing the floor can be found at: https://github.com/misiVoroslakos/3D_printed_designs/blob/main/ThermoMaze/3D_printed_design/cooling_box_v03_platform.stl

## Procedure

- **Calibrating Peltier elements and determining driving voltage**
  1. To determine the ideal driving voltage, grab a single Peltier device, the power supply of the Peltier, two banana plugs with micro grabbers, a thermocouple, and a handheld thermometer.
  2. The Peltier device temperature gradients are chronically stable; so, an assessment of the driving voltage at the experiment's outset would enable consistent temperature drops [9]. The manufacturer recommends using a constant current application to drive the Peltier devices; we adjusted voltage as our power source could generate the necessary constant current based on our constant voltage. We found that the constant current was 0.975A.
**Critical:** For power sources that cannot generate a current of that magnitude, it is prudent to check for and utilize a constant driving current and not voltage.
  3. Connect the banana plug with the micro grabber and turn on the power supply of the Peltier.
  4. Ensure that the experimenter can manipulate the voltage and that the intensity of the current will automatically be adjusted. If your power source does not have the capability to generate a sufficiently high current based on the voltage set, use variable currents and run the following steps adjusting current instead of adjusting voltage.
  5. Set the voltage to 1 V.
  6. Rest the thermocouple on the Peltier device.
  7. Record the initial temperature using the handheld thermometer. The readout should indicate a value around 25 °C (i.e., room temperature).
  8. Connect the micro grabber tips to the cables on the Peltier device.
  9. Use the power supply to drive a current across the Peltier device for a couple of seconds and record the lowest temperature the Peltier achieves.
  10. Remove the micro grabbers from the Peltier cables (breaking the circuit/disconnecting the Peltier element from the power supply), let the Peltier element warm up, and record the highest temperature achieved.
  11. Once back at room temperature, increase the voltage by 0.2 V.
  12. Repeat steps A7–A11 until you identify the target temperature for the study.
  13. We found that stimulating between 2.4 and 3.4V without any heat removal led to our ideal hotspot temperature of 25 °C (Table 1).
*Note: Peltier elements have a built-in temperature gradient (∆T), and so, by switching polarities, the Peltier element will switch from heating to cooling. We found passive cooling via flowing ice water was sufficient to drop the floor temperature to 10 °C; so, we only needed to stimulate the Peltier element to form the hotspot. We identified that ∆T = 10 °C for the Peltier; by flipping the polarity when stimulating, we generated the 35 °C hotspot.*
**Caution:** Each Peltier element has its own ∆T, so it is **imperative to check the specifications and run this type of trial** when starting a project with these devices.
  14. Once the trial is complete (i.e., once the driving voltage is identified), proceed to connect each Peltier element to a cooling block.
    **Table 1.** Trial data for determining driving voltage. Each trial involved measuring the room temperature, the coldest temperature, and the temperature 1 min after the current was stopped (all temperature values measured in °C). Current (in amperes; A) was autogenerated by the power source of the Peltier based on the input voltage (measured in volts; V) of the current generator. *Note: Due to the built-in temperature gradient in the Peltier elements, the difference in temperature is what we focused on, and so it is equally effective to investigate the highest temperature recorded depending on how the element is stimulated.*

Voltage (V)	Current (A)	Initial temperature (°C)	Coldest temperature (°C)	Stable temperature after 1 min (°C)
1.0V	0.300A	22.1°C	19.0°C	22.7°C
1.2V	0.350A	22.1°C	17.9°C	22.6°C
1.4V	0.405A	22.0°C	17.3°C	23.0°C
1.6V	0.480A	22.5°C	17.0°C	23.1°C
1.8V	0.535A	22.2°C	16.3°C	23.4°C
2.0V	0.600A	22.2°C	15.5°C	23.2°C
2.2V	0.650A	22.2°C	15.5°C	23.4°C
2.4V	0.700A	22.4°C	15.3°C	24.5°C
2.6V	0.775A	22.2°C	14.8°C	24.5°C
2.8V	0.820A	22.5°C	14.5°C	24.9°C
3.0V	0.890A	22.5°C	14.3°C	25.2°C
3.2V	0.950A	22.1°C	13.1°C	24.2°C
3.4V	1.000A	22.0°C	12.7°C	24.8°C
3.6V	1.080A	22.2°C	12.4°C	25.4°C
3.8V	1.130A	22.4°C	12.2°C	26.0°C
4.0V	1.200A	22.2°C	12.1°C	25.3°C
4.2V	1.250A	22.4°C	12.0°C	26.5°C
4.4V	1.300A	22.7°C	12.1°C	26.7°C
- **Constructing the floor of the maze**
  1. Take a Peltier device and connect it to the 3D floor printout using hot glue.
  2. Once the glue has hardened, place wood epoxy to fill in any gaps between the Peltier and frame.
  3. After the Peltier is secured to the frame, flip the frame over and ensure that the semi-circle cutouts in the frame (see Figure 1B) align with the locations of the cables on the Peltier.
**Critical:** The Peltier elements will be connected to the power source via banana plugs. It is critical to ensure proper orientation with the cutouts in the frame (Figure 1B) so that all banana plugs will connect to the Peltier while maintaining the levelness of the floor. Failure to properly align the plugs and the cutouts will cause the Peltier to partially pop up out of the frame, creating an unlevel floor.
  4. Once the alignment above is achieved, take one cooling block and orient it such that the flat portion faces the Peltier element.
  5. Place the silver (heat-resistant) epoxy on the flat portion of the cooling block.
  6. Rest the Peltier element on the epoxy until the epoxy hardens; then, cement the Peltier element to the cooling block.
  7. Repeat this process for each of the 25 Peltier devices.
- **Connecting the floor to the water tank**
  1. Once all of the Peltier elements are connected to the cooling blocks and placed in the floor printout, begin working on connecting the water tank to the cooling blocks.
  2. Take the end of a piece of PVC tubing and begin by cutting the pipe into small segments.
  3. Connect the end of one piece of pipe to a submerging DC pump and the other end to the inflow nozzle of element 1.
*Note: The number of the Peltier element used is based on proximity to the water tank, with 1 being the element closest and 5 the element farthest from the water tank.*
  4. After securing the tubing, grab another piece of pipe and secure one end to the outflow nozzle of 1 to the inflow nozzle of 4.
  5. Using separate pieces of pipe, connect the outflow of 4 to the inflow of 2, the outflow of 2 to the inflow of 5, and the outflow of 5 to the inflow of 3.
    **Figure 1.**
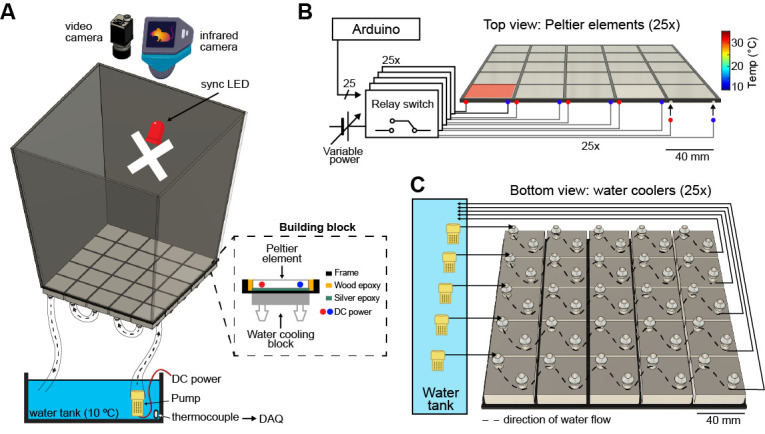
Schematic of the paradigm setup. A) Overview of the maze, video, and IR cameras, LED, and water tank. The dashed box demonstrates the composition of each Peltier element once on the maze floor. B) Connections between the Arduino, relay switches, and Peltier elements. Arrows point to examples of the semicircles cut out of the floor printout to create space for the Peltier’s cables. Red dot: cathode; Blue dot: anode. C) Connections between the water tank and Peltier elements. Yellow squares: water pumps (one pump for each five elements). Dashed lines show the direction of water flow. Reprinted from Vöröslakos et al. [10] with permission.
  6. Finally, take a piece of pipe and connect one end to the outflow of 3 and place the other end into the water tank (see Figure 2D).
  7. Repeat this process for the remaining cooling block elements and submerging DC pumps, ensuring that a single pump circulates the water through one row of Peltier elements.
  8. **Critical:** After **completing all connections** between the **water cooler** and the **five Peltier elements**, check that the **water temperature** arriving at **each Peltier** element is kept cool at the **temperature of the water tank.** There should **be no significant difference—less than 1 °C—between** the **temperature** in the **tank water** and the **water cooling** each of the **five Peltier** elements.
*Note: Our analysis demonstrated that each of the five Peltier elements was efficiently cooled to the tank water temperature.*
  9. Once connections between the floor and water tank are complete, proceed to construct the final part of the ThermoMaze: the walls of the maze.
    **Figure 2.**
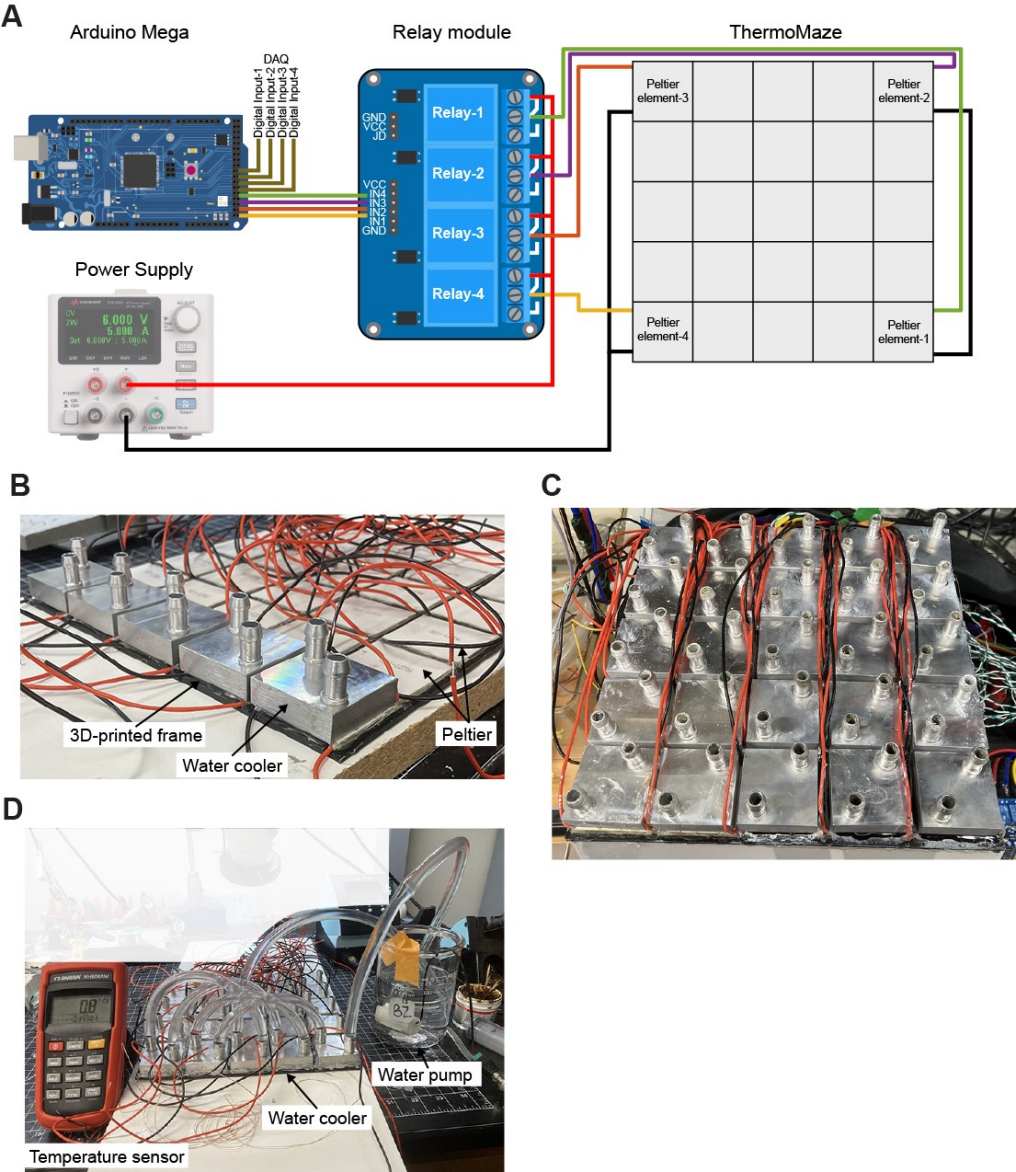
Overview of the connections to run the paradigm. A) Schematic showing the connections between the Arduino mega, the relay module, the hotspots in the ThermoMaze, and the power source of the Peltier elements. B) Photograph of ThermoMaze with all Peltier elements attached to a 3D-printed frame (bottom view). One row of water coolers (n = 5) is also attached to Peltier elements. C) Photograph of the bottom view of the ThermoMaze showing 25 water coolers without tubing attached. D) Demonstration of how the Peltier elements in one row are connected so the water circulates through all the elements. Peltier element 1 is closest to the pump and 5 is closest to the thermometer when determining the tubing connections between Peltier elements (steps C3–6). This example demonstrates that passive cooling suffices to drop the surface temperature of the Peltier elements to the desired value, as the ice-cold water flowing through the tubes and between the water coolers and Peltier elements (turned off) passively reduced the surface temperature of the Peltier element to 0.8 °C. The temperature is measured by a K-type thermocouple attached to the surface of the last Peltier element in a row. Figure is reprinted from Vöröslakos et al. [10] with permission.
- **Constructing the box**
  1. To construct the walls, take the cast acrylic and cut out four identical walls measuring 20 cm × 20 cm × 40 cm (width × length × height).
  2. Using hot glue, secure the first wall to the floor.
*Note: Hot glue was used so that the walls could be angled outward, providing a large opening on top for the camera to easily visualize the entirety of the maze.*
  3. After securing that wall in place, take the second wall and secure both to the floor of the maze and to the first wall using hot glue.
  4. Repeat this process with the remaining two walls to complete the construction of the ThermoMaze box.
- **Connecting the Peltier elements to the power supply of the Peltier**
  1. Open the Arduino code for the behavioral paradigm.
  2. Connect the Arduino mega board to the computer and transfer the behavioral code to the board.
  3. Connect the Arduino board to a relay module.
  4. Connect each of the four Peltier elements that will serve as hotspots to each of the four relay elements in the module.
  5. Connect the positive side of the variable voltage source to each of the four relay modules, and the negative side of the variable voltage source to each of the four Peltier elements that will be hotspots.
*Note: To enable the single port (on the variable voltage source) to connect the four other parts (either the relay spots or the hotspots), stack banana plugs so that all four plugs can access a single spot.*
- **Hotspot customization**
  1. All aspects of the hotspots (location, order, duration) are customizable and should be modified to the experiment's needs.
  2. Each Peltier element is individually connected to the relay module, allowing any of the 25 Peltier elements to serve as a hotspot, and providing the experimenter with the option to have anywhere from 1 to 25 hotspots. To adjust the hotspot location, alter the connections between the relay module and Peltier elements such that the code will activate the newly chosen hotspots (in Figure 2A, moving the colored connections to different elements will shift the hotspots to the new locations).
  3. To adjust the order of the hotspots, edit the Arduino code ( https://github.com/misiVoroslakos/3D_printed_designs/blob/main/ThermoMaze/ThermoMaze_control/Cooling_box_v04_relays_20220410.ino ) and reorder when each relay and TTL signal are high (see lines 4–16 and 69–120 of the code).
  4. Increasing the duration of each hotspot allows for sleep at said spot. We found that turning hotspots on for periods of 20 min was sufficient for triggering sleep [10]. To alter the duration of the hotspot, change the number in line 27 of the above code (can be compared to https://github.com/misiVoroslakos/3D_printed_designs/blob/main/ThermoMaze/ThermoMaze_control/Cooling_box_v04_relays_20220410_sleep.ino ).
- **Final touches in constructing the paradigm**
  1. Tape an Arduino-compatible LED roughly halfway up one of the walls of the maze (Figure 1A). In our construction, the LED was taped approximately 20 cm up the wall and roughly centered on the wall.
**Critical:** Ensure the LED is high enough on the wall that the animal cannot reach it.
*Notes:*
    - *The LED serves two purposes: 1) the pulsing of the LED enables you to sync the video to the electrophysiology signals, so that should any frames drop, the LED will provide clear timing between the signals as to when the frames dropped; and 2) the LED serves as a distal cue.*
    - *In placing the LED, the coordinates do not need to be measured precisely. Rather, by eye the LED should be roughly halfway up and in the center of the wall. Ensure that the LED is blinking and visualized on both the IR and video cameras.*
  2. Secure both the IR camera and the video camera above the maze.
  3. Calibrate the IR camera.
    - On the IR camera, click on settings, and select the feature that enables you to change the temperature gradient.
    - With the gradient adjustable, **press *lock* on the upper bound** while the lower bound remains adjustable.
    - **Pause point:** Ensure that the upper bound is locked and the lower bound is adjustable. If both bounds are adjustable, the pre-existing range will not change, but the bounds on the range will change in unison. If both are locked, then no changes will occur.
    - Set the lower temperature bound to a value well below the lowest expected floor temperature (for our purposes, the lower bound was set to 4 °C).
    - **Lock the lower bound** and **unlock the upper bound** so that it is adjustable.
    - **Pause point:** Ensure the lower bound is locked and the upper bound is adjustable. See step G3c above for further explanation.
    - Set the upper temperature bound to a value well above the highest expected floor temperature (for our purposes, the upper bound was set to 40 °C).
    - **Lock the upper bound**.
    - **Critical:** Ensure that both bounds are locked at the desired values before proceeding. With the bounds set on the IR camera, confirm that it is connected to the computer.
    - On the computer, open the Windows built-in video recorder and check that the IR camera captures the entire field.
    - The IR camera is now properly calibrated.
  4. Calibrate the video camera.
    - To begin calibrating the video camera, ensure that the LED is turned on (i.e., blinking).
    - On the computer, open the Intan RHD USB board interface software.
    - Check that the LED pulse signal is visible.
    - Once you confirm the LED pulses are picked up by the Intan RHD USB board interface software, open the pylon viewer software.
    - Confirm that the entirety of the maze is clearly displayed on the screen.
    - The video camera is now properly calibrated.
  5. Prepare the pulley system for freely moving recordings.
    - To enable extracellular electrophysiological recordings while the animal is in the maze, set up a pulley system.
    - The system should have a long, wire-thin cable with a connector to a head stage on the end.
    - Once the implanted probe is connected to the head stage during an experimental session, adjust the slack in the long wire-thin cable. This cable should not be too loose that it folds over itself and affects the mouse's movement, and not so tight that it pulls up on the mouse’s head.
  6. Once the maze is constructed and you finish all connections, check for any electromagnetic interference (EMI)-induced noise.
**Critical:** Checks for EMI-induced noise should be conducted upon completing construction (to ensure no faulty connections).
    - To check for EMI noise, place an implanted animal inside the ThermoMaze and run the “Cooling” sub-session protocol.
    - If line noise (50/60 Hz) appears on all recording channels regardless of the location of the animal, then the ground connection of the submerging pump(s) is improper.
*Note: Tap water includes impurities, such as dissolved sodium, calcium, and magnesium salts; thus, it is an excellent conductor of electricity.*
    - If line noise (50/60 Hz) appears on all recording channels when the animal is approaching an active Peltier, then the ground connection of that Peltier is improper.
*Note: Peltier effect produces a temperature difference between two sides of a Peltier device when a current is flowing. Improper grounding of Peltier device(s) can induce spatially restricted line noise.*
  7. Potential solutions to remedy any EMI-induced noise identified:
    - Ensure that all wires are tightly screwed into their location.
    - Avoid ground loops and make sure that all electronic devices (Peltier devices, submerging pumps, Arduino, relays, and recording system) share a common ground.
*Note: Use a multimeter to test the continuity of the ground lines.*
    - For any noise stemming from the submerging pumps, remove all pumps from the water tank. Place one pump inside the water tank and observe the brain signal for potential line noise. If there is no electrical noise, repeat these steps with the remaining submerging pumps.
**Critical:** Replace any submerging pump(s) that induces electrical noise. If noise still persists, consider using deionized pure water instead of tap water.
    - Check for any moveable appliances (which could be the source of the noise); remove any and all of these appliances from the recording room.
    - Try moving the wires from the relay system around to see if there is a position that removes the noise.
    - For any noise stemming from the long wire-thin cable, try to move it around. Once you find a position that keeps the signal noise-free, rest the cable on a nearby platform or clip it to a nearby surface.
    - Wrap some of the cables in aluminum foil, which can help to re-ground the signal. For this option, continue to test different groupings of wires until you find a place where the aluminum foil wrapped around the wires removes the EMI noise.
    - If none of these solutions remedy the EMI-induced noise, check all cables for signs of damage or fraying. Replace the cables and wires if necessary.
- **Running the paradigm (Figure 3A)**
  **Figure 3.**
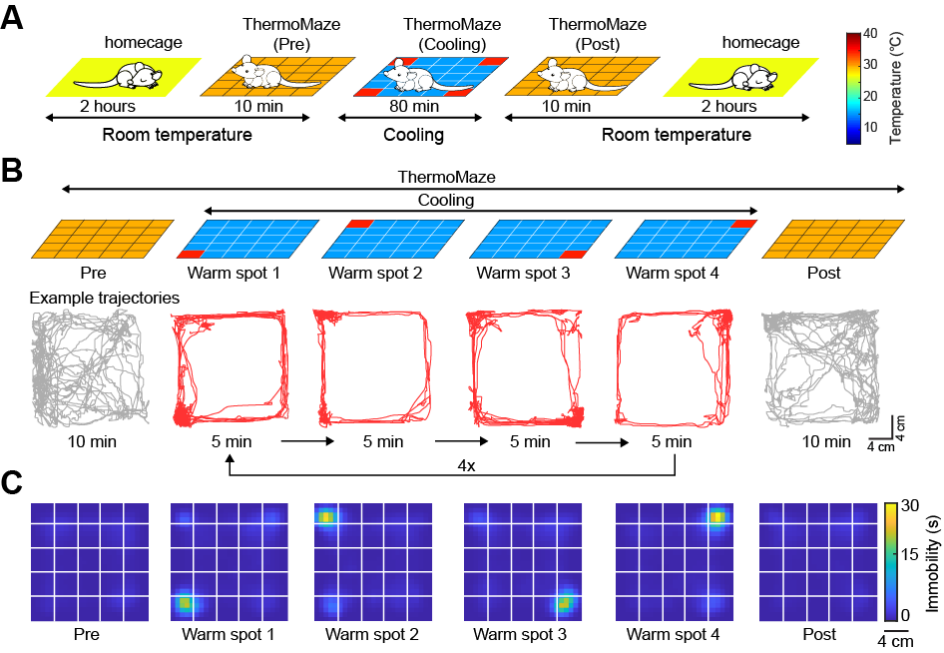
Overview of the paradigm. A) Five sub-sessions constitute a daily recording session: (i) rest epoch in the home cage, (ii) pre-cooling exploration epoch (Pre), (iii) cooling, (iv) post-cooling exploration epoch (Post), and (v) another rest in the home cage. B) Schematic of temperature landscape changes when the animal is in the ThermoMaze (top) and example animal trajectory (below). During cooling, one Peltier element always provides a warm spot for the animal (four Peltier elements in the four corners were used in this experiment). Each Peltier element was turned on for 5 min in a sequential order (1–2–3–4) and the sequence was repeated four times. C) Session-averaged duration of immobility (speed ≤ 2.5 cm/s) that the animal spent at each location in the ThermoMaze. Color code: temporal duration of immobility(s); white lines divide the individual Peltier elements; n = 17 sessions in 7 mice. Adapted from Vöröslakos et al. [10] with permission.
  1. Pre-maze home cage baseline recording (2 h; Figure 3Ai)
    - Begin by placing the mouse (small rodent) in a home cage in the same room as the maze.
*Note: The animal has access to food or water throughout the duration of the home-cage experimental session.*
    - Connect the mouse to the recording software and check for any EMI-induced noise. Follow procedure step G7 to remedy any EMI-induced noise that should arise.
    - Once the signal is free of EMI-induced noise, start the recording.
    - To avoid any olfactory cues from affecting behavior, the experimenter should either leave or remain in the recording room for the entire 2 h.
    - After 2 h, stop the recording and keep the mouse in the home cage as you set up the maze.
  2. Pre-behavior in maze baseline (*Pre-cooling*; 10 min; Figure 3Aii)
    - Fill the tank halfway with 25 °C water.
    - **Critical:** Ensure that all five submerged pumps are completely covered. If they are not completely submerged, their vibrations can generate EMI noise and serve as a noxious auditory stimulus to the rodent.
    - Ensure the LED light is securely taped to the wall of the maze and is blinking.
    - Take the mouse from its home cage and place it in the maze, ensuring that the rodent either remains connected to or is reconnected to the recording software once in the maze. If the **mouse is anxious** after placement **in the maze** and ensuring connections to the recording software, **allow it** a bit of time **to calm down** before proceeding to the next step.
    - Turn on the pumps to begin cycling the water on the floor of the maze.
    - **Critical:** Place a **finger** onto the **suction point** of each of the five submerged **pumps** and check that each is working properly (you should **feel the suction pulling on your finger**). If there are any suction issues, detach the tube from the pump (allowing water to flow through the pump) and reattach them to remedy the issue.
    - For those using tap water, ensure there is no EMI-induced noise in the signal. If noise is present in the signal, it is likely due to one of the pumps. Remedy the issue by gently resting the problematic pump on a neighboring tube (light enough that it does not obstruct flow) to remove the effect of the pump vibrating against the floor of the tank. Using distilled water is unlikely to cause any issues as it is free of conductive ions.
    - **Critical:** Start the electrophysiological recording, then the IR and regular video recordings. The **electrophysiological recording must be started prior to the video recordings** to ensure that **all video frames** are **timestamped** to a data point in the **electrical recording**, so that if there are any issues with the video recordings (e.g., dropped frames), their points can be identified on the electrical data.
    - After 10 min, stop the IR and regular video recording and then the electrophysiological recording.
    - **Critical: Stop** the **video** recordings while **still collecting electrophysiological data** (see reasoning in step H2h).
    - Once all recordings are stopped, turn off the pumps.
  3. Run the paradigm (*Cooling*; 80 min; Figure 3Aiii, Video 1)
    - With the pumps stopped (and the water no longer circulating), fill the tank with crushed ice to lower the temperature to 10 °C using the handheld thermometer to continuously monitor the temperature. Make sure to **stir** the **ice** around the tank **before checking temperature** to ensure the water temperature is uniform.
    - Once at 10 °C, place two reusable ice packs (“ice blocks”) to stabilize the temperature at 10 °C.
*Note: The ice blocks will keep the temperature stabilized for the duration of the cooling portion of the experiment (80 min) in standard laboratory conditions (room temperature kept at 21 ).*
    - Once the water temperature is stable, ensure the LED is still blinking, check for any EMI-induced noise, and remedy any issues (see steps F6 and F7).
    - **Critical: Start** the **electrophysiological recording** and, **subsequently**, begin the IR and regular **video recording**. See step G2h for the importance of starting electrical recordings prior to video recordings.
    - Once the electrophysiological and video recordings have begun, ensure the driving voltage is properly set on the Peltier driving power supply, begin circulating the water, and start the behavioral paradigm software protocol.
    - **Critical: Ensure** that the **pumps** and **the behavioral paradigm** are **working properly**. To check the pumps and solve any issues, see step G2f. To ensure the paradigm is working properly, pull up the Windows built-in video recording software on the computer. The entire floor should be at the temperature of the circulating water, except for one hotspot, which should be at 35 °C. Also, check that the animal finds and remains immobile on the hotspot.
    - As the paradigm is running, the experimenter should remain in the room and sporadically check all of the maze parameters (i.e., check for any EMI-induced noise, water temperature, and IR video).
    - If any EMI-induced noise is identified, follow step F7 to resolve the issue.
    - If the water temperature exceeds 13.5 °C, **add** some more **crushed ice** in **small increments**, stirring the water (with your finger) and then checking the temperature after each increment. **Do not let** the **water temperature** fall **below 9 °C**.
    - When checking the video, ensure the floor is at the water temperature (10 °C) except for the hotspot (which should be at 35 °C), that the animal is finding and remaining immobile on the hotspot, and that you see the hotspot switch locations.
    **Video 1.**
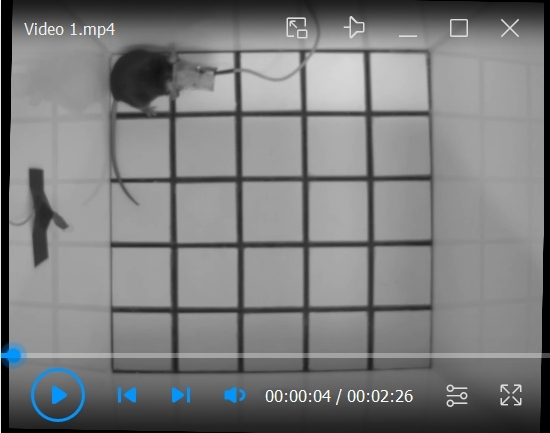
Mouse engaging in the ThermoMaze behavioral task. Adapted from Vöröslakos et al. [10] with permission.
  4. Post-behavior in-maze baseline (*Post-cooling*; 10 min; Figure 3Aiv)
    - After 80 min, the behavioral paradigm should be complete. Immediately stop circulating the water (to help the floor start to warm up for the animal), then stop the IR and regular video recordings, and finally stop the electrophysiology recording (see Procedure G2h for the importance of stopping order).
    - Once all of the recordings are stopped, remove the ice blocks from the tank and pour out some of the tank water into a bucket (making room for adding warmer water).
    - Add warm/hot water to the tank to raise the water temperature back to 25 °C.
    - Once the water is at 25 °C, **immediately begin circulating** the water to make the rodent more comfortable.
    - Once the pumps are turned on, follow steps H2h–k.
  5. Post-maze home cage baseline recording (2 h; Figure 3Av)
    - With all of the recordings stopped, take the rodent out of the maze and place it back in the home cage where the pre-maze home cage baseline recording occurred.
*Note: The animal has access to food and water during the duration of the home-cage experimental session.*
    - Connect the mouse to the recording software and check for any EMI-induced noise. Follow step F7 to remedy any EMI-induced noise that should arise.
    - Once the signal is free of EMI-induced noise, start the recording.
    - To avoid any olfactory cues from affecting behavior, the experimenter should either leave or remain in the recording room for the entire 2 h.
    - After 2 h of recording, the experimental session is now complete.

## Data analysis

Detailed description of data analysis can be found in the *Quantification and statistical analysis* section of Vöröslakos et al. [10]. To perform the analysis, expertise using MATLAB is necessary.

## Validation of protocol

**Figure 4.**
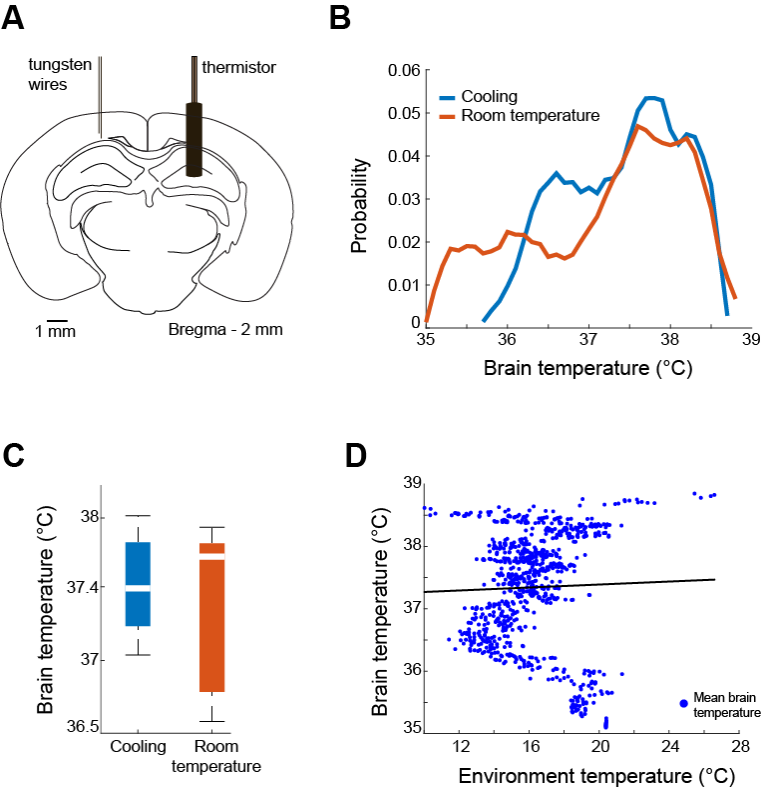
Effects of environmental temperature on brain temperature. A) Schematic of the thermistor and tungsten wire implantation for trials comparing brain temperature changes during cooling and room temperature sessions. B) Probability mass function of brain temperature during cooling (blue) and room temperature (orange) sub-sessions. The data came from 10 behavioral trials using two mice. The graph demonstrates that in both cooling and ambient temperature conditions, the hippocampal temperature stayed within a physiological range. C) Median brain temperature during both cooling (blue) and ambient (orange) conditions. There is no significant difference in brain temperature when cooling vs. ambient temperature conditions (Kolmogorov-Smirnov test). D) Linear regression demonstrating a lack of correlation between brain temperature and environmental temperature (R = 0.03, p = 0.384). Adapted from Vöröslakos et al. [10] with permission.

The preparations and data analysis are exactly as described previously [10] and are reprinted below for convenience. All experiments were approved by the Institutional Animal Care and Use Committee at New York University Langone Medical Center. Animals were handled daily and accommodated to the experimenter and the ThermoMaze before surgery and electrophysiological recordings. Mice (adult female n = 8, mean = 22 g; male n = 5, mean = 26 g) were kept in a vivarium on a 12/12 h light/dark cycle and housed two per cage before surgery and individually after it. Atropine (0.05 mg/kg, s.c.) was administered after isoflurane anesthesia induction to reduce saliva production. Body temperature was kept between 36 and 37 °C via a temperature controller (TCAT-LV; Physitemp, Clifton, NJ). Stages of anesthesia were maintained by confirming the lack of a nociceptive reflex. The skin of the head was shaved, and the surface of the skull was cleaned by hydrogen peroxide (2%). A custom 3D-printed baseplate ([11]; Form2 printer, FormLabs, Sommerville, MA) was attached to the skull using C&B Metabond dental cement (Parkell, Edgewood, NY). The craniotomy site was marked and a stainless-steel ground screw was placed above the cerebellum. Silicon probe attached to a metal Microdrive [12] was implanted into the dorsal CA1 of the hippocampus (Bregma: -2 mm AP, -1.5 mm ML). For surgeries testing brain temperature changes, tungsten wires were implanted in place of the silicone probe, and a thermistor was placed in the contralateral dorsal CA1 (Bregma: -2 mm AP, +1.5 mm ML; Figure 4A). A protective copper mesh cap was built around the probe. Animals received ketoprofen (5.2 mg/kg, s.c.) at the end of the surgery and on each of the following two days and were given at least five days to recover prior to experiments. The electrophysiology data was digitized at 20,000 samples/s using an RHD2000 recording system (Intan technologies, Los Angeles, CA). All data analyses occurred using custom codes in MATLAB.

**Hippocampal temperature unaffected**

The next concern to address is validating the paradigm-assessed brain temperature. Homeostatic thermoregulation is critical to ensure proper physiological functioning. Mice brain temperatures operate within a 4 °C range (35.5–39.5 °C), with fluctuations arising based on behavioral state [13]. Temperatures below this range can affect the time course of action potentials and neurotransmitter release [14].

Rodents rely on environmental temperature for thermoregulation. Since this paradigm capitalizes on rodent thermotaxis and thermoregulatory mechanisms, we needed to ensure that the experimenter-generated abrasive environmental temperature (10 °C on non-hotspots) did not cause temperature deviations outside of the physiologic range. To assuage this concern, we implanted mice (n = 2) with thermistors at the same coordinates contralaterally to the recording probes (see Figure 4A) and ran the paradigm collecting information on brain temperature. Tungsten wires were placed in place of silicon probes to monitor electrical activity. The hippocampal temperature remained within the thermoregulatory zone, albeit with a shift toward the zone’s upper bounds. This finding confirms and reaffirms the dependency of brain temperature on behavioral state [13,15,16] and suggests tightly regulated mechanisms for homeostatic temperature maintenance, which are independent of the ambient temperature [10].

**Paradigm generates rapid and uniform temperature changes**

**Figure 5.**
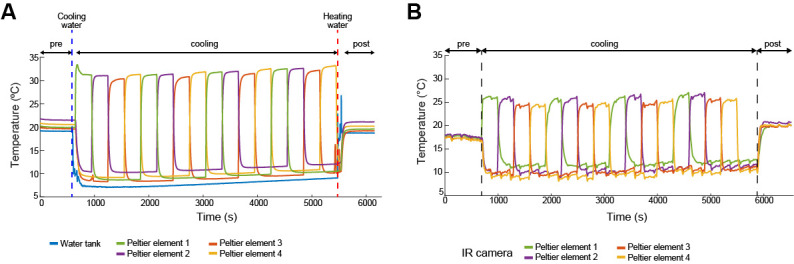
Temperature changes during the animal-free emulated behavioral sessions. A) Temperature changes in each hotspot measured via thermocouples. Each color corresponds to a Peltier element. Results demonstrate the floor temperature dropped to the water tank temperature within 15 s of the pumps turning on, except for the hotspot, which increased to 35 °C. The transition in hotspot location (both the heating of the new and cooling of the old hotspots) occurred within 30 s. B) Same as A), but temperatures were measured via IR camera. Adapted from Vöröslakos et al. [10] with permission.

Initial validation of the protocol began with confirmation that the code for generating hotspots ( https://github.com/misiVoroslakos/3D_printed_designs/blob/main/ThermoMaze/ThermoMaze_control/Cooling_box_v04_relays_20220410.ino ) led to rapid and uniform temperature changes in the floor. To accomplish this, we ran an emulated behavioral session without an animal. A thermocouple was placed in each of the four hotspots, the water was cooled to 10 °C (experimental conditions), and the behavioral portion of the paradigm was run (step H3). We found that cooling of the floor was uniform, and the transition between heating spots occurred within 30 s (i.e., after each 5 min period, there were 30 s as the prior hotspot cooled down and the new hotspot warmed up to 35 °C; Figure 5).

**Electrophysiological and behavioral changes during cooling**

**Figure 6.**
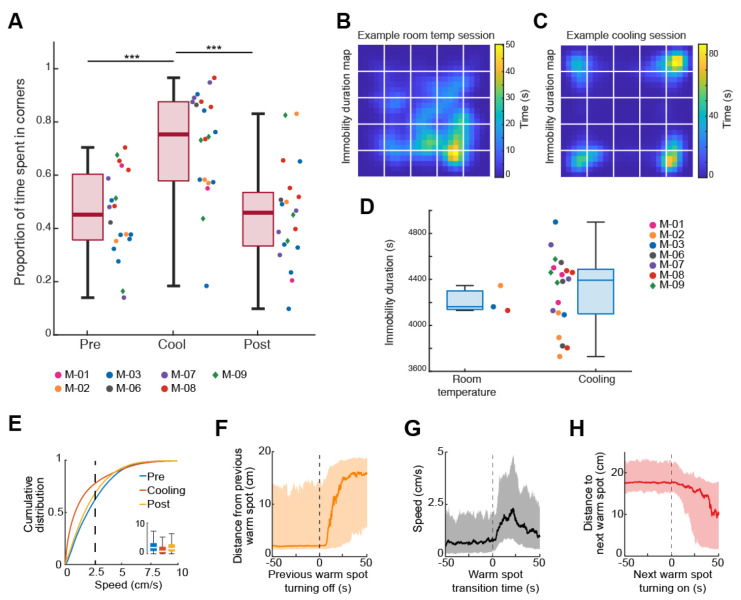
Cooling generated periods of immobility in the hotspots. A) Comparison between the pre-cooling, cooling, and post-cooling of the proportion of time mice spent in any of the corners (hotspots) of the maze. Median, Kruskal–Wallis test: H = 19.69, d.f. = 2, p = 5.29 × 10^-5^. The proportion of time spent in any corner during cooling was significantly greater than during either pre-cooling (p = 0.0004) or post-cooling (p = 0.0003). The proportion of time spent in any corner during pre- and post-cooling did not significantly differ (p = 0.9996). Dots (females) and diamonds (males) between the boxes represent the individual sessions, and the same color represents sessions from the same animal. B) Immobility duration map of an example session in which the animal was in the ThermoMaze under 25 °C (room temperature) condition. Immobility spatial distribution demonstrates that, during ambient conditions, the SPW-R are centered around the mouse’s preferred corner. C) Same as B) but during a cooling session. Distribution demonstrates that during cooling, the immobility is focused on the hotspots. D) Immobility durations within an 80-min period of free exploration of the ThermoMaze either under room temperature or during the cooling sub-session in two groups of mice (room temperature n = 3; cooling n = 20; p = 0.49, one-sided Wilcoxon rank sum tests). E) Cumulative distribution of animal speed in the ThermoMaze during three sub-sessions from 7 mice). Median, Kruskal–Wallis test: H = 139304.10, d.f. = 2, p < 0.001. F) Animal’s distance from the previously heated Peltier element site. G) Speed of the animal centered around warm spot transitions. H) Animal’s distance from the target warm spot as a function of time (red curve: median; time 0 = onset of heating). Modified from Vöröslakos et al. [10] with permission.

Initial investigations revolved around assessing the effects of the maze in shaping behavior, learning that the paradigm posed little issue to the mice, as it took only a few practice sessions before they readily found the hotspot (median = 3 practice sessions). Subsequently, we looked at how the cooling shaped behavior. As theorized, the cold environment promoted immobility (Figure 6), as the mice spent a smaller proportion of time moving during cooling compared to pre- and post-cooling (cooling: 23 ± 12%, pre-cooling: 40 ± 19%, post-cooling: 34 ± 16%, mean ± SD, movement defined as speed > 2.5 cm/s). Conversely, the mice spent a greater percentage of time immobile during cooling compared to pre- and post-cooling (cooling: 76.74 ± 12.41%, pre-cooling: 59 ± 19%, post-cooling: 66 ± 16%, mean ± SD, immobility defined as speed < 2.5 cm/s).

Changing hotspot locations triggered exploration (Figure 6E–H). Video 1 shows the thermotaxis behavior of a mouse in the ThermoMaze (infra-red image is overlaid on the raw video, the second half of the video is 10 times faster than real time). The mice hastily transitioned from immobility to movement (median = 12.28 s, n = 20 sessions across 7 mice; transition is defined as the time taken from the hotspot shutting off for the mice to increase their speed from 0 to 2.5 cm/s), promptly abandoning their location (median = 12.99 s, n = 20 sessions across 7 mice), and finding the new hotspot (median = 23.45 s, n = 20 sessions across 7 mice).

**Neuronal firing during sharp wave ripples demonstrates place-selectivity at experimenter-designated locations**

**Figure 7.**
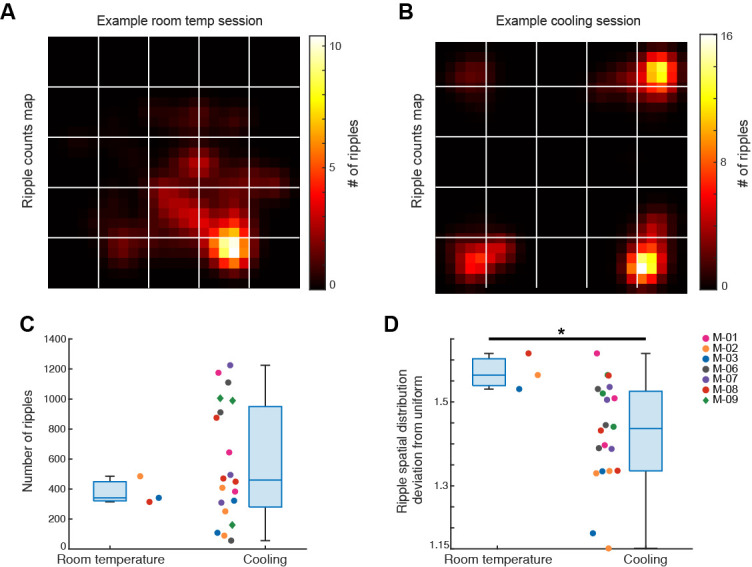
Cooling demonstrated place-specific firing during sharp wave ripples (SPW-Rs). A) Heat map showing SPW-R counts during an example session in which the animal was in the ThermoMaze under 25 °C room temperature condition. The SPW-R spatial distribution demonstrates that, during ambient conditions, the SPW-R is centered around the mouse’s preferred corner. B) Heat map showing SPW-R counts during an example cooling session. The map demonstrates that during cooling, the SPW-R firing was restricted to the hotspots. C) Total SPW-R counts within an 80-min period of free exploration of the ThermoMaze either under room temperature or during the cooling sub-session in two groups of mice (room temperature n = 3; cooling n = 20; p = 0.62, one-sided Wilcoxon rank sum test). D) Same plots as in C) but for the degree to which their spatial distributions deviate from a uniform distribution (p = 0.04, one-sided Wilcoxon rank sum test). Modified from Vöröslakos et al. [10] with permission.

Brain states underlying behaviors can dichotomously split into “preparative” or “consummatory” [17], where the preparative class relates to ongoing appetitive behaviors that culminate in consummatory behavior. Consummatory behaviors are sometimes termed non-voluntary or non-conscious states and can be identified via electrophysiological monitoring of various brain states [18]. Of particular note, sharp wave ripples (SPW-Rs) in the hippocampus are a hallmark of consummatory behaviors [19]. SPW-Rs are critical to spatial memory processes [20–23]. They are hypothesized to participate in sleep consolidation processes by transmitting spiking information from the hippocampus to the neocortex as temporally compressed packets of the spiking that occurred while awake [24]. SPW-Rs most frequently fire during non-rapid eye movement (NREM) sleep or periods of awake immobility, both of which characterize the major behavioral state of the mice during cooling. Given that SPW-Rs readily appear during NREM sleep and awake immobility and that they are critical to consolidating spatial information, our electrophysiological assessment focused on neuronal firing within SPW-Rs. We found that pyramidal (excitatory) neurons demonstrated greater place-specific firing compared to interneurons (inhibitory), and both classes showed a higher place-specific firing compared to controls (one-sided Wilcoxon rank sum test, p < 0.001; Figure 7).

**Discussion**

We developed the ThermoMaze, a new small rodent behavioral paradigm that is a non-aqueous Morris water maze analog. The paradigm forces the animal into long periods of immobility at experimenter-chosen locations, allowing electrophysiological analysis of the immediate changes in neural activity. The paradigm relies on the use of innate thermotaxis behaviors, leading the rodent to find and stay immobile in a heated area while the ambient temperature remains cold. Utilization of natural thermotaxis mechanisms enhances the ethological validity of the findings, an important feature in designing behavioral paradigms for animals [4].

One potential issue that arose is that the current design of the ThermoMaze is relatively small (20 cm × 20 cm). Traditional open fields for mice measure 40 cm × 40 cm. Slight modifications to the 3D floor design ( https://github.com/misiVoroslakos/3D_printed_designs/blob/main/ThermoMaze/3D_printed_design/cooling_box_v03_platform.stl ) and to the cast acrylic cut will enable the maze to be scaled to bigger dimensions. Additionally, the current size leads the animal to follow the walls to each of the four hotspots (located at the corners) and rarely visit the central portion of the maze. We found that altering hotspot location leads the mice to find the new hotspot away from a corner, providing better spatial coverage of the maze [10]. Given small rodents' aversion to large, open spaces, this will pose a problem irrespective of maze size.

The reliance on innate behavioral mechanisms led the rodents to rapidly learn the task (i.e., finding the hotspot), requiring only three practice sessions on average before an animal was completely trained. Additionally, the paradigm does not alter brain temperatures, signifying that the electrophysiological data represents naturally occurring changes and not some brain temperature–dependent changes. The place-specific firing patterns of neurons within sharp wave ripples, both inhibitory (interneurons) and excitatory (pyramidal cells), indicate that many consolidation processes occur in the periods of immobility immediately following a behavior. Therefore, utilization of this paradigm will enable researchers to gather large data sets on processes that have yet to be thoroughly investigated.

To facilitate widespread use of the paradigm, we provide detailed descriptions of the construction process, along with links for purchasing all necessary equipment. We further include links to the 3D-printable elements and relevant codes, accessible on the Buzsaki Lab GitHub ( https://github.com/misiVoroslakos/3D_printed_designs/tree/main/ThermoMaze ). We hope that this helps to foster the use of the ThermoMaze, a non-aqueous analog of the Morris water maze.
